# 3-*epi*-Dammarenediol II 1.075 hydrate: a dammarane triterpene from the bark of *Aglaia eximia*

**DOI:** 10.1107/S1600536812040366

**Published:** 2012-10-10

**Authors:** Hoong-Kun Fun, Suchada Chantrapromma, Asep Supriadin, Desi Harneti, Unang Supratman

**Affiliations:** a X-ray Crystallography Unit, School of Physics, Universiti Sains Malaysia, 11800 USM, Penang, Malaysia; b Department of Pharmaceutical Chemistry, College of Pharmacy, King Saud University, PO Box 2457, Riyadh 11451, Saudi Arabia; c Crystal Materials Research Unit, Department of Chemistry, Faculty of Science, Prince of Songkla University, Hat-Yai, Songkhla 90112, Thailand; d Department of Chemistry, Faculty of Mathematics and Natural Sciences, Padjadjaran University, Jatinangor 45363, West Java, Indonesia

## Abstract

The title dammarane tritepene, 3α,20(S)-dihy­droxy­dammar-24-ene, which crystallized out in a hydrated form, C_30_H_52_O_2_.1.075H_2_O, was isolated from the *Aglaia eximia* bark. The three cyclo­hexane rings adopt chair conformations. The cyclo­pentane has an envelope conformation with the quaternary C at position 14 as the flap atom with the maximum deviation of 0.288 (2) Å. The methyl­heptene side chain is disordered over two positions with 0.505 (1):0.495 (1) site occupancies and is axially attached with an (+)-*syn*-*clinal* conformation. The hydroxyl group at position 3 of dammarane is in a different conformation to the corresponding hydroxyl in Dammarenediol II. In the crystal, the dammarane and water mol­ecules are linked by O_Dammarane_—H⋯O_water_ and O_water_—H⋯O_Dammarane_ hydrogen bonds into a three-dimensional network.

## Related literature

For ring conformations, see: Cremer & Pople (1975 ▶). For bond-length data, see: Allen *et al.* (1987 ▶). For background to *Aglaia* plants, triterpenoids and their biological activity, see: Asakawa *et al.* (1977 ▶); Chairgulprasert *et al.* (2006 ▶); Greger *et al.* (2001 ▶); Grosvenor *et al.* (1995 ▶); Lima *et al.* (2004 ▶); Qiu *et al.* (2001 ▶); Roux *et al.* (1998 ▶); Yodsaoue *et al.* (2012 ▶); Zhang *et al.* (2010 ▶). For related structures, see: Qiu *et al.* (2001 ▶). For the stability of the temperature controller used in the data collection, see Cosier & Glazer (1986 ▶). 
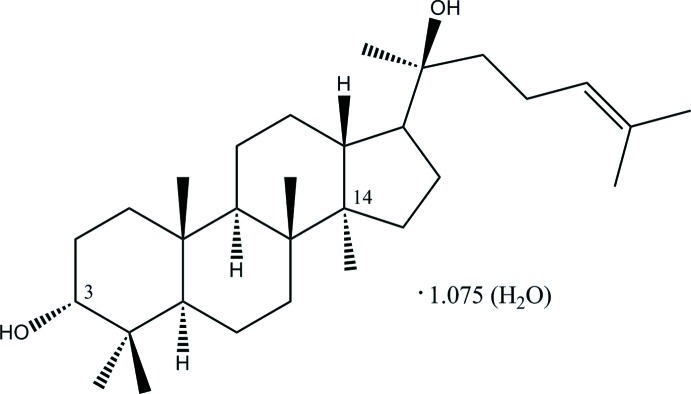


## Experimental

### 

#### Crystal data

C_30_H_52_O_2_·1.075H_2_O
*M* *_r_* = 463.99
Tetragonal, 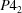

*a* = 19.9481 (13) Å
*c* = 7.3410 (7) Å
*V* = 2921.2 (5) Å^3^
*Z* = 4
Mo *K*α radiation
μ = 0.07 mm^−1^
*T* = 100 K
0.39 × 0.11 × 0.10 mm

#### Data collection

Bruker APEX Duo CCD area-detector diffractometer
Absorption correction: multi-scan (*SADABS*; Bruker, 2009 ▶) *T*_min_ = 0.975, *T*_max_ = 0.994
24864 measured reflections
4543 independent reflections
3887 reflections with *I* > 2σ(*I*)
*R*_int_ = 0.064

#### Refinement

*R*[*F* ^2^ > 2σ(*F* ^2^)] = 0.058
*wR*(*F* ^2^) = 0.162
*S* = 1.07
4543 reflections
332 parameters
1 restraint
H-atom parameters constrained
Δρ_max_ = 0.72 e Å^−3^
Δρ_min_ = −0.48 e Å^−3^

### 

Data collection: *APEX2* (Bruker, 2009 ▶); cell refinement: *SAINT* (Bruker, 2009 ▶); data reduction: *SAINT* (Bruker, 2009 ▶); program(s) used to solve structure: *SHELXTL* (Sheldrick, 2008 ▶); program(s) used to refine structure: *SHELXTL* (Sheldrick, 2008 ▶); molecular graphics: *SHELXTL* (Sheldrick, 2008 ▶); software used to prepare material for publication: *SHELXTL* (Sheldrick, 2008 ▶) and *PLATON* (Spek, 2009 ▶).

## Supplementary Material

Crystal structure: contains datablock(s) global, I. DOI: 10.1107/S1600536812040366/bv2209sup1.cif

Additional supplementary materials:  crystallographic information; 3D view; checkCIF report

## Figures and Tables

**Table 1:** Hydrogen-bond geometry (Å, °)

*D*—H⋯*A*	*D*—H	H⋯*A*	*D*⋯*A*	*D*—H⋯*A*
O2—H1*O*2⋯O1*W*	0.84	2.02	2.816 (3)	157
O1*W*—H1*W*1⋯O1^i^	0.84	1.94	2.783 (3)	175
O2*W*—H1*W*2⋯O2^ii^	0.83	1.89	2.718 (3)	177

Symmetry codes: (i) 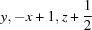; (ii) 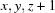.

**Fig. 1.**
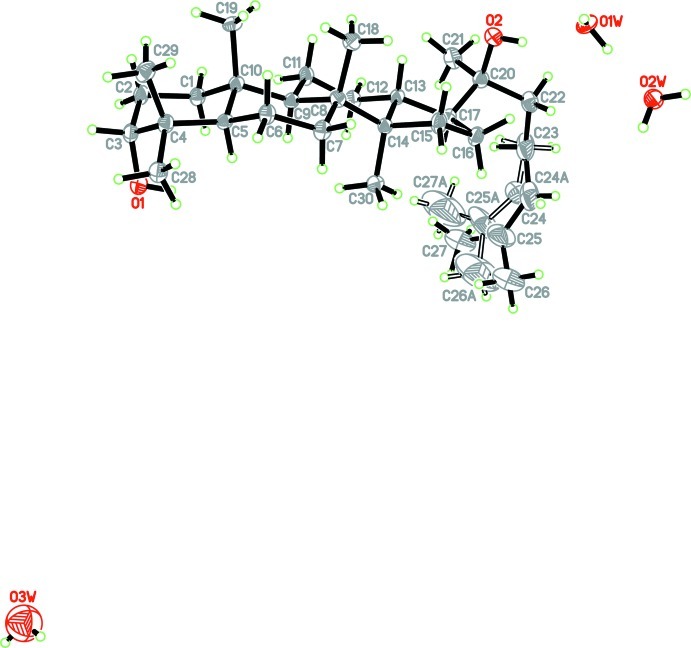
The molecular structure of the title compound, showing 60% probability displacement ellipsoids and the atom-numbering scheme. One H atom each of the O1W and O2W water molecules was generated by a symmetry operation -x, -y, z. Open bonds show the minor component.

**Fig. 2.**
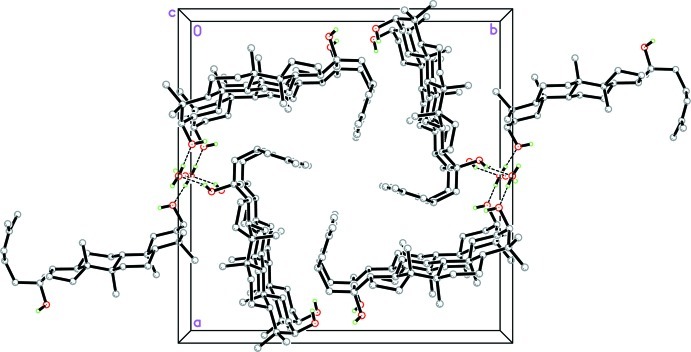
The crystal packing of the major component viewed along the c axis, only hydroxyl H and H atoms involving in hydrogen bonds are shown for clarity. O—H···O hydrogen bonds were drawn as dashed lines.

## supplementary crystallographic information

### Comment

*Aglaia* genus plants belonging to the Mahogany family have been known as a good source of organic acids, sesquiterpenes, diterpenes and triterpenes (Chairgulprasert *et al.*, 2006; Qiu *et al.*, 2001; Roux *et al.*, 1998; Yodsaoue *et al.*, 2012). Many of the various terpenenoids from this genus possess interesting biological properties such as anti-inflammatory (Yodsaoue *et al.*, 2012), cytotoxic (Zhang *et al.*, 2010) and insecticidal (Greger *et al.*, 2001) activities. The title compound (I), 3-*epi*-Dammarenediol II or 3*α*,20(S)-dihydroxydammar-24-ene, was previously isolated from *Trattinnickia burserifolia* (Lima *et al.*, 2004). However it is now isolated for the first time from *Aglaia eximia*, a plant which was used as a traditional medicine for the treatment of malaria in Indonesia (Grosvenor *et al.*, 1995). Herein the crystal structure of (I) is reported.

Compound (I) has a dammarane nucleus and crystallized in a hydrated form, C_30_H_52_O_2_.1.075(H_2_O) (Fig. 1). Two of the water molecules, O1W and O2W, have half occupancies and lie on two-fold axis, the other H of each water molecule was generated by a symmetry operation, -x, -y, z, whereas the third water molecule, O3W, has 0.075 occupancy. The molecule of dammarane has four fused rings and all rings are in *trans*-fused conformation. The three cyclohexane rings are in standard chair conformations. The cyclopentane (C13–C17) adopts an envelope conformation with the puckered C14 atom having the maximum deviation of 0.288 (2) Å, Q = 0.457 (3) Å and θ = 220.1 (3)° (Cremer & Pople, 1975). The hydroxyl group at atom C3 is axially attached which is different from the corresponding hydroxyl group in Dammarenediol II (Asakawa *et al.*, 1977). The methylheptene side chain is disordered over two positions; the major component and the minor component *A* (Fig. 1), with the refined site-occupancy ratio of 0.505 (1)/0.495 (1) and is axially attached at atom C20 with the torsion angle of C17–C20–C22–C23 = 58.83 (3)°, indicating an (+)-syn-clinal conformation with respect to the cyclopentane ring (Fig. 1). The bond distances in (I) are within normal ranges (Allen *et al.*, 1987) and comparable to a related structure (Qiu *et al.*, 2001).

The crystal packing of (I) is consolidated by intermolecular O_Dammarane_—H···O_water_ and O_water_—H···O_Dammarane_ hydrogen bonds (Table 1). The molecules of 3-*epi*-Dammarenediol II and water molecules are linked by O—H···O hydrogen bonds into a three dimensional network (Fig. 2).

### Experimental

The dried and milled bark of *A. eximia* (3 kg) which was collected from Bogor Botanical Garden, West Java, Indonesia, was extracted successively by n-hexane, ethyl acetate and methanol at room temperature. The ethyl acetate extract (300 g) was subjected to vacuum chromatography on silica gel G 60 by using a step gradient of n-hexane-ethyl acetate methanol. The fraction eluted by n-hexane/ethyl acetate (3:2) was further separated by column chromatography on silica gel (chloroform: methanol; 9.5:0.5 v/v) to give a colorless solid (63 mg) of the title compound. Colorless needle-shaped single crystals of the title compound suitable for X-ray structure determination were recrystallized from ethyl acetate at room temperature after several days.

### Refinement

One of the water molecules, O3W, was refined isotropically. H atoms were positioned geometrically and allowed to ride on their parent atoms, with d(O—H) = 0.83-0.84 Å, d(C—H) = 1.00 Å for cyclic CH, 0.95 for CH, 0.99 for CH_2_ and 0.98 Å for CH_3_ atoms. The *U*_iso_ values were constrained to be 1.2*U*_eq_ of the carrier atom for all H atoms. A rotating group model was used for the methyl groups. A total of 3923 Friedel pairs were merged before final refinement. The methylheptene side chain is disordered over two sites with refined site occupancies of 0.505 (1) and 0.495 (1). The same U_ij_ parameters were used for atom pairs C23/C24, C26/C27 and C26A/C27A. A number of reflections were omitted from the final refinement owing to poor agreement.

### Crystal data

**Table:**

C_30_H_52_O_2_·1.075H_2_O	*D*_x_ = 1.055 Mg m^−^^3^
*M**_r_* = 463.99	Mo *K*α radiation, λ = 0.71073 Å
Tetragonal, *P*4_2_	Cell parameters from 4543 reflections
Hall symbol: P 4c	θ = 2.0–30.0°
*a* = 19.9481 (13) Å	µ = 0.07 mm^−^^1^
*c* = 7.3410 (7) Å	*T* = 100 K
*V* = 2921.2 (5) Å^3^	Needle, colourless
*Z* = 4	0.39 × 0.11 × 0.10 mm
*F*(000) = 1035	

### Data collection

**Table:**

Bruker APEX Duo CCD area-detector diffractometer	4543 independent reflections
Radiation source: sealed tube	3887 reflections with *I* > 2σ(*I*)
Graphite monochromator	*R*_int_ = 0.064
φ and ω scans	θ_max_ = 30.0°, θ_min_ = 2.0°
Absorption correction: multi-scan (*SADABS*; Bruker, 2009)	*h* = −28→27
*T*_min_ = 0.975, *T*_max_ = 0.994	*k* = −22→28
24864 measured reflections	*l* = −10→10

### Refinement

**Table:**

Refinement on *F*^2^	Secondary atom site location: difference Fourier map
Least-squares matrix: full	Hydrogen site location: inferred from neighbouring sites
*R*[*F*^2^ > 2σ(*F*^2^)] = 0.058	H-atom parameters constrained
*wR*(*F*^2^) = 0.162	*w* = 1/[σ^2^(*F*_o_^2^) + (0.1014*P*)^2^ + 0.4141*P*] where *P* = (*F*_o_^2^ + 2*F*_c_^2^)/3
*S* = 1.07	(Δ/σ)_max_ < 0.001
4543 reflections	Δρ_max_ = 0.72 e Å^−^^3^
332 parameters	Δρ_min_ = −0.48 e Å^−^^3^
1 restraint	Absolute structure: nd
Primary atom site location: structure-invariant direct methods	

### Special details

**Table:**

Experimental. The crystal was placed in the cold stream of an Oxford Cryosystems Cobra open-flow nitrogen cryostat (Cosier & Glazer, 1986) operating at 100.0 (1) K.
Geometry. All esds (except the esd in the dihedral angle between two l.s. planes) are estimated using the full covariance matrix. The cell esds are taken into account individually in the estimation of esds in distances, angles and torsion angles; correlations between esds in cell parameters are only used when they are defined by crystal symmetry. An approximate (isotropic) treatment of cell esds is used for estimating esds involving l.s. planes.
Refinement. Refinement of F^2^ against ALL reflections. The weighted R-factor wR and goodness of fit S are based on F^2^, conventional R-factors R are based on F, with F set to zero for negative F^2^. The threshold expression of F^2^ > 2sigma(F^2^) is used only for calculating R-factors(gt) etc. and is not relevant to the choice of reflections for refinement. R-factors based on F^2^ are statistically about twice as large as those based on F, and R- factors based on ALL data will be even larger.

### Fractional atomic coordinates and isotropic or equivalent isotropic displacement parameters (Å^2^)

**Table:**

	*x*	*y*	*z*	*U*_iso_*/*U*_eq_	Occ. (<1)
O1	0.40685 (8)	0.04934 (8)	0.2210 (3)	0.0194 (4)	
H1O1	0.3988	0.0883	0.2587	0.023*	
O2	0.09002 (8)	0.45212 (8)	0.2073 (3)	0.0202 (4)	
H1O2	0.0731	0.4721	0.2969	0.024*	
C1	0.30479 (11)	0.12044 (11)	0.0082 (4)	0.0158 (4)	
H1A	0.2901	0.1373	−0.1122	0.019*	
H1B	0.3454	0.1457	0.0439	0.019*	
C2	0.32266 (12)	0.04584 (12)	−0.0089 (3)	0.0175 (5)	
H2A	0.2832	0.0208	−0.0543	0.021*	
H2B	0.3593	0.0404	−0.0987	0.021*	
C3	0.34456 (12)	0.01670 (11)	0.1731 (4)	0.0160 (4)	
H3A	0.3534	−0.0323	0.1566	0.019*	
C4	0.29152 (12)	0.02521 (11)	0.3236 (4)	0.0155 (4)	
C5	0.27016 (11)	0.10018 (11)	0.3325 (3)	0.0133 (4)	
H5A	0.3118	0.1245	0.3697	0.016*	
C6	0.21933 (12)	0.11581 (11)	0.4842 (4)	0.0164 (4)	
H6A	0.1741	0.1007	0.4466	0.020*	
H6B	0.2319	0.0914	0.5965	0.020*	
C7	0.21811 (12)	0.19144 (11)	0.5223 (4)	0.0164 (4)	
H7A	0.2623	0.2051	0.5715	0.020*	
H7B	0.1840	0.2007	0.6170	0.020*	
C8	0.20235 (11)	0.23448 (11)	0.3524 (3)	0.0135 (4)	
C9	0.24808 (11)	0.21167 (10)	0.1905 (3)	0.0132 (4)	
H9A	0.2946	0.2217	0.2330	0.016*	
C10	0.24882 (11)	0.13407 (11)	0.1492 (3)	0.0137 (4)	
C11	0.23870 (12)	0.25678 (11)	0.0224 (4)	0.0164 (4)	
H11A	0.1925	0.2511	−0.0247	0.020*	
H11B	0.2702	0.2423	−0.0742	0.020*	
C12	0.25087 (12)	0.33151 (11)	0.0648 (4)	0.0167 (4)	
H12A	0.2982	0.3385	0.1008	0.020*	
H12B	0.2418	0.3589	−0.0450	0.020*	
C13	0.20444 (11)	0.35310 (11)	0.2196 (3)	0.0142 (4)	
H13A	0.1576	0.3429	0.1800	0.017*	
C14	0.21829 (11)	0.31048 (11)	0.3922 (3)	0.0136 (4)	
C15	0.17269 (12)	0.34658 (12)	0.5301 (4)	0.0185 (5)	
H15A	0.1845	0.3342	0.6567	0.022*	
H15B	0.1249	0.3357	0.5083	0.022*	
C16	0.18674 (13)	0.42168 (11)	0.4941 (4)	0.0183 (5)	
H16A	0.2245	0.4376	0.5703	0.022*	
H16B	0.1466	0.4490	0.5218	0.022*	
C17	0.20495 (12)	0.42675 (11)	0.2870 (4)	0.0156 (4)	
H17A	0.2518	0.4442	0.2769	0.019*	
C18	0.12704 (11)	0.22514 (12)	0.3090 (4)	0.0183 (5)	
H18A	0.1002	0.2512	0.3956	0.022*	
H18B	0.1153	0.1776	0.3190	0.022*	
H18C	0.1179	0.2408	0.1849	0.022*	
C19	0.18212 (12)	0.10878 (12)	0.0669 (4)	0.0185 (5)	
H19A	0.1909	0.0699	−0.0113	0.022*	
H19B	0.1616	0.1446	−0.0054	0.022*	
H19C	0.1516	0.0958	0.1654	0.022*	
C20	0.15827 (12)	0.47476 (11)	0.1821 (4)	0.0166 (5)	
C21	0.17162 (13)	0.47347 (13)	−0.0233 (4)	0.0217 (5)	
H21A	0.1578	0.4300	−0.0731	0.026*	
H21B	0.2196	0.4803	−0.0458	0.026*	
H21C	0.1460	0.5093	−0.0824	0.026*	
C22	0.16225 (13)	0.54729 (12)	0.2552 (4)	0.0219 (5)	
H22A	0.1308	0.5752	0.1838	0.026*	
H22B	0.1462	0.5472	0.3828	0.026*	
C23	0.23106 (15)	0.58110 (14)	0.2509 (7)	0.0405 (9)	
H23A	0.2262	0.6298	0.2290	0.049*	0.505 (14)
H23B	0.2590	0.5618	0.1526	0.049*	0.505 (14)
H23C	0.2503	0.5715	0.1294	0.049*	0.495 (14)
H23D	0.2227	0.6300	0.2552	0.049*	0.495 (14)
C24	0.2660 (5)	0.5675 (4)	0.4513 (17)	0.0405 (9)	0.505 (14)
H24A	0.2382	0.5748	0.5545	0.049*	0.505 (14)
C25	0.3285 (5)	0.5474 (5)	0.486 (2)	0.053 (3)	0.505 (14)
C26	0.3527 (5)	0.5366 (8)	0.680 (2)	0.083 (4)	0.505 (14)
H26A	0.3176	0.5503	0.7651	0.100*	0.505 (14)
H26B	0.3931	0.5634	0.7008	0.100*	0.505 (14)
H26C	0.3631	0.4890	0.6978	0.100*	0.505 (14)
C27	0.3782 (5)	0.5330 (7)	0.339 (2)	0.083 (4)	0.505 (14)
H27A	0.3692	0.4886	0.2867	0.100*	0.505 (14)
H27B	0.4237	0.5339	0.3894	0.100*	0.505 (14)
H27C	0.3744	0.5670	0.2429	0.100*	0.505 (14)
C24A	0.2789 (4)	0.5695 (4)	0.3779 (19)	0.041 (3)	0.495 (14)
H24B	0.2655	0.5767	0.5006	0.050*	0.495 (14)
C25A	0.3409 (4)	0.5498 (5)	0.355 (3)	0.073 (5)	0.495 (14)
C26A	0.3867 (5)	0.5364 (7)	0.512 (3)	0.111 (6)	0.495 (14)
H26D	0.3610	0.5374	0.6262	0.133*	0.495 (14)
H26E	0.4217	0.5708	0.5165	0.133*	0.495 (14)
H26F	0.4074	0.4922	0.4977	0.133*	0.495 (14)
C27A	0.3696 (5)	0.5328 (8)	0.159 (3)	0.111 (6)	0.495 (14)
H27D	0.4145	0.5521	0.1466	0.133*	0.495 (14)
H27E	0.3400	0.5517	0.0662	0.133*	0.495 (14)
H27F	0.3720	0.4840	0.1444	0.133*	0.495 (14)
C28	0.32341 (12)	0.00442 (12)	0.5058 (4)	0.0188 (5)	
H28A	0.3460	−0.0389	0.4914	0.023*	
H28B	0.2884	0.0006	0.5990	0.023*	
H28C	0.3561	0.0384	0.5433	0.023*	
C29	0.23313 (13)	−0.02388 (12)	0.2882 (4)	0.0212 (5)	
H29A	0.2497	−0.0701	0.2956	0.025*	
H29B	0.2146	−0.0157	0.1666	0.025*	
H29C	0.1981	−0.0170	0.3800	0.025*	
C30	0.29122 (12)	0.32099 (12)	0.4601 (4)	0.0188 (5)	
H30A	0.3048	0.3675	0.4376	0.023*	
H30B	0.3213	0.2906	0.3945	0.023*	
H30C	0.2936	0.3115	0.5909	0.023*	
O1W	0.0000	0.5000	0.4726 (4)	0.0189 (5)	
H1W1	0.0159	0.5297	0.5419	0.023*	
O2W	0.0000	0.5000	0.9617 (4)	0.0187 (5)	
H1W2	0.0278	0.4839	1.0342	0.022*	
O3W	0.967 (2)	0.016 (2)	0.858 (7)	0.069 (12)*	0.07
H1W3	0.9518	0.0223	0.9645	0.083*	0.07
H2W3	1.0084	0.0055	0.8469	0.083*	0.07

### Atomic displacement parameters (Å^2^)

**Table:**

	*U*^11^	*U*^22^	*U*^33^	*U*^12^	*U*^13^	*U*^23^
O1	0.0211 (8)	0.0185 (8)	0.0188 (9)	0.0018 (6)	−0.0003 (7)	−0.0031 (7)
O2	0.0176 (8)	0.0214 (8)	0.0215 (10)	0.0016 (6)	−0.0002 (7)	−0.0016 (8)
C1	0.0201 (10)	0.0153 (9)	0.0119 (11)	0.0026 (8)	0.0011 (9)	0.0000 (9)
C2	0.0245 (11)	0.0172 (10)	0.0108 (11)	0.0040 (8)	0.0002 (9)	−0.0032 (9)
C3	0.0209 (10)	0.0133 (9)	0.0139 (11)	0.0034 (8)	−0.0001 (9)	−0.0017 (8)
C4	0.0196 (10)	0.0134 (9)	0.0136 (11)	0.0006 (8)	0.0001 (9)	−0.0001 (9)
C5	0.0160 (10)	0.0150 (9)	0.0089 (10)	0.0004 (7)	−0.0002 (8)	−0.0010 (8)
C6	0.0202 (10)	0.0152 (9)	0.0139 (12)	0.0008 (8)	0.0035 (9)	0.0002 (9)
C7	0.0219 (10)	0.0170 (10)	0.0103 (10)	0.0013 (8)	0.0028 (9)	0.0012 (9)
C8	0.0143 (9)	0.0163 (10)	0.0098 (10)	0.0021 (7)	0.0006 (8)	0.0009 (8)
C9	0.0155 (9)	0.0135 (9)	0.0106 (11)	0.0013 (7)	−0.0004 (8)	−0.0006 (8)
C10	0.0157 (10)	0.0145 (9)	0.0109 (11)	0.0007 (7)	0.0000 (8)	−0.0009 (8)
C11	0.0220 (10)	0.0162 (10)	0.0109 (11)	0.0038 (8)	0.0015 (9)	0.0000 (9)
C12	0.0210 (11)	0.0164 (10)	0.0127 (11)	0.0025 (8)	0.0024 (9)	0.0022 (9)
C13	0.0168 (9)	0.0146 (9)	0.0112 (11)	0.0022 (7)	−0.0013 (9)	−0.0018 (9)
C14	0.0166 (10)	0.0139 (9)	0.0101 (10)	0.0032 (8)	−0.0011 (8)	−0.0015 (8)
C15	0.0244 (11)	0.0179 (10)	0.0133 (11)	0.0044 (8)	0.0029 (9)	−0.0017 (9)
C16	0.0264 (11)	0.0153 (10)	0.0131 (11)	0.0045 (8)	−0.0025 (10)	−0.0025 (9)
C17	0.0181 (10)	0.0135 (9)	0.0151 (11)	0.0013 (7)	−0.0005 (9)	−0.0029 (9)
C18	0.0156 (10)	0.0197 (10)	0.0197 (12)	−0.0001 (8)	0.0013 (9)	−0.0025 (10)
C19	0.0183 (10)	0.0205 (10)	0.0167 (12)	−0.0009 (8)	−0.0063 (9)	−0.0021 (9)
C20	0.0185 (10)	0.0153 (10)	0.0161 (12)	0.0019 (8)	0.0001 (9)	0.0010 (9)
C21	0.0266 (12)	0.0228 (11)	0.0157 (12)	0.0060 (9)	0.0029 (10)	0.0043 (10)
C22	0.0238 (11)	0.0149 (10)	0.0272 (15)	0.0023 (8)	0.0022 (10)	0.0007 (10)
C23	0.0309 (14)	0.0176 (11)	0.073 (3)	−0.0046 (9)	0.0144 (16)	−0.0107 (14)
C24	0.0309 (14)	0.0176 (11)	0.073 (3)	−0.0046 (9)	0.0144 (16)	−0.0107 (14)
C25	0.024 (4)	0.047 (4)	0.090 (9)	−0.003 (3)	−0.013 (5)	−0.006 (5)
C26	0.040 (4)	0.102 (6)	0.108 (9)	0.019 (4)	−0.029 (4)	−0.032 (6)
C27	0.040 (4)	0.102 (6)	0.108 (9)	0.019 (4)	−0.029 (4)	−0.032 (6)
C24A	0.021 (3)	0.022 (3)	0.081 (8)	−0.006 (2)	−0.006 (4)	−0.007 (4)
C25A	0.017 (4)	0.036 (4)	0.166 (17)	−0.003 (3)	0.001 (6)	0.007 (7)
C26A	0.035 (4)	0.091 (6)	0.207 (18)	−0.008 (3)	−0.016 (6)	0.018 (8)
C27A	0.035 (4)	0.091 (6)	0.207 (18)	−0.008 (3)	−0.016 (6)	0.018 (8)
C28	0.0238 (11)	0.0178 (10)	0.0149 (11)	0.0024 (8)	0.0000 (10)	0.0012 (9)
C29	0.0261 (12)	0.0150 (10)	0.0224 (13)	−0.0030 (9)	−0.0012 (10)	−0.0004 (10)
C30	0.0198 (10)	0.0167 (10)	0.0197 (12)	0.0005 (8)	−0.0056 (10)	−0.0016 (9)
O1W	0.0233 (12)	0.0190 (11)	0.0145 (12)	−0.0064 (9)	0.000	0.000
O2W	0.0190 (11)	0.0212 (11)	0.0158 (12)	0.0021 (9)	0.000	0.000

### Geometric parameters (Å, º)

**Table:**

O1—C3	1.446 (3)	C18—H18C	0.9800
O1—H1O1	0.8400	C19—H19A	0.9800
O2—C20	1.446 (3)	C19—H19B	0.9800
O2—H1O2	0.8400	C19—H19C	0.9800
C1—C2	1.535 (3)	C20—C21	1.531 (4)
C1—C10	1.547 (3)	C20—C22	1.545 (3)
C1—H1A	0.9900	C21—H21A	0.9800
C1—H1B	0.9900	C21—H21B	0.9800
C2—C3	1.522 (3)	C21—H21C	0.9800
C2—H2A	0.9900	C22—C23	1.530 (4)
C2—H2B	0.9900	C22—H22A	0.9900
C3—C4	1.539 (3)	C22—H22B	0.9900
C3—H3A	1.0000	C23—C24A	1.354 (11)
C4—C28	1.538 (4)	C23—C24	1.650 (13)
C4—C29	1.544 (3)	C23—H23A	0.9900
C4—C5	1.557 (3)	C23—H23B	0.9900
C5—C6	1.538 (3)	C23—H23C	0.9900
C5—C10	1.565 (3)	C23—H23D	0.9899
C5—H5A	1.0000	C24—C25	1.335 (13)
C6—C7	1.535 (3)	C24—H24A	0.9500
C6—H6A	0.9900	C25—C27	1.496 (19)
C6—H6B	0.9900	C25—C26	1.516 (19)
C7—C8	1.546 (3)	C26—H26A	0.9800
C7—H7A	0.9900	C26—H26B	0.9800
C7—H7B	0.9900	C26—H26C	0.9800
C8—C18	1.547 (3)	C27—H27A	0.9800
C8—C9	1.566 (3)	C27—H27B	0.9800
C8—C14	1.577 (3)	C27—H27C	0.9800
C9—C11	1.539 (3)	C24A—C25A	1.309 (12)
C9—C10	1.577 (3)	C24A—H24B	0.9500
C9—H9A	1.0000	C25A—C26A	1.50 (3)
C10—C19	1.546 (3)	C25A—C27A	1.58 (3)
C11—C12	1.542 (3)	C26A—H26D	0.9800
C11—H11A	0.9900	C26A—H26E	0.9800
C11—H11B	0.9900	C26A—H26F	0.9800
C12—C13	1.528 (3)	C27A—H27D	0.9800
C12—H12A	0.9900	C27A—H27E	0.9800
C12—H12B	0.9900	C27A—H27F	0.9800
C13—C17	1.550 (3)	C28—H28A	0.9800
C13—C14	1.551 (3)	C28—H28B	0.9800
C13—H13A	1.0000	C28—H28C	0.9800
C14—C15	1.540 (3)	C29—H29A	0.9800
C14—C30	1.552 (3)	C29—H29B	0.9800
C15—C16	1.547 (3)	C29—H29C	0.9800
C15—H15A	0.9900	C30—H30A	0.9800
C15—H15B	0.9900	C30—H30B	0.9800
C16—C17	1.566 (4)	C30—H30C	0.9800
C16—H16A	0.9900	O1W—H1W1	0.8422
C16—H16B	0.9900	O2W—H1W2	0.8324
C17—C20	1.542 (3)	O3W—O3W^i^	1.45 (9)
C17—H17A	1.0000	O3W—H1W3	0.8496
C18—H18A	0.9800	O3W—H2W3	0.8504
C18—H18B	0.9800		
			
C3—O1—H1O1	109.4	C20—C17—C13	115.2 (2)
C20—O2—H1O2	109.3	C20—C17—C16	112.63 (19)
C2—C1—C10	113.14 (19)	C13—C17—C16	104.30 (19)
C2—C1—H1A	109.0	C20—C17—H17A	108.2
C10—C1—H1A	109.0	C13—C17—H17A	108.2
C2—C1—H1B	109.0	C16—C17—H17A	108.2
C10—C1—H1B	109.0	C8—C18—H18A	109.5
H1A—C1—H1B	107.8	C8—C18—H18B	109.5
C3—C2—C1	111.4 (2)	H18A—C18—H18B	109.5
C3—C2—H2A	109.3	C8—C18—H18C	109.5
C1—C2—H2A	109.3	H18A—C18—H18C	109.5
C3—C2—H2B	109.3	H18B—C18—H18C	109.5
C1—C2—H2B	109.3	C10—C19—H19A	109.5
H2A—C2—H2B	108.0	C10—C19—H19B	109.5
O1—C3—C2	106.76 (19)	H19A—C19—H19B	109.5
O1—C3—C4	111.5 (2)	C10—C19—H19C	109.5
C2—C3—C4	113.01 (19)	H19A—C19—H19C	109.5
O1—C3—H3A	108.5	H19B—C19—H19C	109.5
C2—C3—H3A	108.5	O2—C20—C21	106.5 (2)
C4—C3—H3A	108.5	O2—C20—C17	108.10 (19)
C28—C4—C3	108.07 (19)	C21—C20—C17	112.1 (2)
C28—C4—C29	106.7 (2)	O2—C20—C22	107.25 (18)
C3—C4—C29	109.2 (2)	C21—C20—C22	110.4 (2)
C28—C4—C5	109.61 (19)	C17—C20—C22	112.2 (2)
C3—C4—C5	108.92 (18)	C20—C21—H21A	109.5
C29—C4—C5	114.21 (19)	C20—C21—H21B	109.5
C6—C5—C4	113.93 (19)	H21A—C21—H21B	109.5
C6—C5—C10	110.83 (18)	C20—C21—H21C	109.5
C4—C5—C10	116.96 (19)	H21A—C21—H21C	109.5
C6—C5—H5A	104.5	H21B—C21—H21C	109.5
C4—C5—H5A	104.5	C23—C22—C20	116.9 (2)
C10—C5—H5A	104.5	C23—C22—H22A	108.1
C7—C6—C5	109.98 (19)	C20—C22—H22A	108.1
C7—C6—H6A	109.7	C23—C22—H22B	108.1
C5—C6—H6A	109.7	C20—C22—H22B	108.1
C7—C6—H6B	109.7	H22A—C22—H22B	107.3
C5—C6—H6B	109.7	C24A—C23—C22	122.9 (5)
H6A—C6—H6B	108.2	C22—C23—C24	106.7 (4)
C6—C7—C8	113.7 (2)	C24A—C23—H23A	110.4
C6—C7—H7A	108.8	C22—C23—H23A	110.4
C8—C7—H7A	108.8	C24—C23—H23A	110.4
C6—C7—H7B	108.8	C24A—C23—H23B	92.3
C8—C7—H7B	108.8	C22—C23—H23B	110.4
H7A—C7—H7B	107.7	C24—C23—H23B	110.4
C7—C8—C18	107.26 (19)	H23A—C23—H23B	108.6
C7—C8—C9	109.42 (17)	C24A—C23—H23C	108.3
C18—C8—C9	112.0 (2)	C22—C23—H23C	106.4
C7—C8—C14	110.1 (2)	C24—C23—H23C	127.4
C18—C8—C14	110.48 (18)	H23A—C23—H23C	94.6
C9—C8—C14	107.62 (18)	C24A—C23—H23D	105.4
C11—C9—C8	111.60 (17)	C22—C23—H23D	106.4
C11—C9—C10	114.9 (2)	C24—C23—H23D	101.8
C8—C9—C10	115.88 (18)	H23B—C23—H23D	120.1
C11—C9—H9A	104.3	H23C—C23—H23D	106.5
C8—C9—H9A	104.3	C25—C24—C23	127.9 (9)
C10—C9—H9A	104.3	C25—C24—H24A	116.1
C19—C10—C1	107.6 (2)	C23—C24—H24A	116.1
C19—C10—C5	115.41 (19)	C24—C25—C27	122.6 (12)
C1—C10—C5	107.65 (18)	C24—C25—C26	121.3 (12)
C19—C10—C9	112.78 (18)	C27—C25—C26	116.1 (8)
C1—C10—C9	107.94 (18)	C25A—C24A—C23	129.1 (13)
C5—C10—C9	105.14 (19)	C25A—C24A—H24B	115.5
C9—C11—C12	112.6 (2)	C23—C24A—H24B	115.5
C9—C11—H11A	109.1	C24A—C25A—C26A	122.3 (17)
C12—C11—H11A	109.1	C24A—C25A—C27A	121.4 (14)
C9—C11—H11B	109.1	C26A—C25A—C27A	116.1 (11)
C12—C11—H11B	109.1	C25A—C26A—H26D	109.5
H11A—C11—H11B	107.8	C25A—C26A—H26E	109.5
C13—C12—C11	109.10 (19)	H26D—C26A—H26E	109.5
C13—C12—H12A	109.9	C25A—C26A—H26F	109.5
C11—C12—H12A	109.9	H26D—C26A—H26F	109.5
C13—C12—H12B	109.9	H26E—C26A—H26F	109.5
C11—C12—H12B	109.9	C25A—C27A—H27D	109.5
H12A—C12—H12B	108.3	C25A—C27A—H27E	109.5
C12—C13—C17	120.0 (2)	H27D—C27A—H27E	109.5
C12—C13—C14	110.19 (18)	C25A—C27A—H27F	109.5
C17—C13—C14	104.92 (19)	H27D—C27A—H27F	109.5
C12—C13—H13A	107.0	H27E—C27A—H27F	109.5
C17—C13—H13A	107.0	C4—C28—H28A	109.5
C14—C13—H13A	107.0	C4—C28—H28B	109.5
C15—C14—C13	100.11 (17)	H28A—C28—H28B	109.5
C15—C14—C30	106.2 (2)	C4—C28—H28C	109.5
C13—C14—C30	110.8 (2)	H28A—C28—H28C	109.5
C15—C14—C8	116.91 (19)	H28B—C28—H28C	109.5
C13—C14—C8	109.86 (19)	C4—C29—H29A	109.5
C30—C14—C8	112.24 (17)	C4—C29—H29B	109.5
C14—C15—C16	103.5 (2)	H29A—C29—H29B	109.5
C14—C15—H15A	111.1	C4—C29—H29C	109.5
C16—C15—H15A	111.1	H29A—C29—H29C	109.5
C14—C15—H15B	111.1	H29B—C29—H29C	109.5
C16—C15—H15B	111.1	C14—C30—H30A	109.5
H15A—C15—H15B	109.0	C14—C30—H30B	109.5
C15—C16—C17	105.70 (19)	H30A—C30—H30B	109.5
C15—C16—H16A	110.6	C14—C30—H30C	109.5
C17—C16—H16A	110.6	H30A—C30—H30C	109.5
C15—C16—H16B	110.6	H30B—C30—H30C	109.5
C17—C16—H16B	110.6	O3W^i^—O3W—H1W3	113.1
H16A—C16—H16B	108.7	H1W3—O3W—H2W3	118.4
			
C10—C1—C2—C3	−57.8 (3)	C11—C12—C13—C14	59.5 (2)
C1—C2—C3—O1	−66.0 (2)	C12—C13—C14—C15	173.65 (18)
C1—C2—C3—C4	56.9 (3)	C17—C13—C14—C15	43.1 (2)
O1—C3—C4—C28	−50.8 (2)	C12—C13—C14—C30	61.8 (2)
C2—C3—C4—C28	−171.06 (19)	C17—C13—C14—C30	−68.7 (2)
O1—C3—C4—C29	−166.47 (19)	C12—C13—C14—C8	−62.8 (2)
C2—C3—C4—C29	73.3 (2)	C17—C13—C14—C8	166.71 (17)
O1—C3—C4—C5	68.2 (2)	C7—C8—C14—C15	−68.6 (2)
C2—C3—C4—C5	−52.0 (3)	C18—C8—C14—C15	49.7 (3)
C28—C4—C5—C6	−59.4 (3)	C9—C8—C14—C15	172.24 (19)
C3—C4—C5—C6	−177.4 (2)	C7—C8—C14—C13	178.30 (18)
C29—C4—C5—C6	60.3 (3)	C18—C8—C14—C13	−63.4 (2)
C28—C4—C5—C10	169.14 (19)	C9—C8—C14—C13	59.1 (2)
C3—C4—C5—C10	51.1 (3)	C7—C8—C14—C30	54.5 (3)
C29—C4—C5—C10	−71.2 (3)	C18—C8—C14—C30	172.8 (2)
C4—C5—C6—C7	162.2 (2)	C9—C8—C14—C30	−64.6 (3)
C10—C5—C6—C7	−63.4 (2)	C13—C14—C15—C16	−44.8 (2)
C5—C6—C7—C8	56.5 (3)	C30—C14—C15—C16	70.5 (2)
C6—C7—C8—C18	72.7 (2)	C8—C14—C15—C16	−163.3 (2)
C6—C7—C8—C9	−49.0 (3)	C14—C15—C16—C17	30.3 (2)
C6—C7—C8—C14	−167.05 (18)	C12—C13—C17—C20	86.8 (3)
C7—C8—C9—C11	−175.21 (18)	C14—C13—C17—C20	−148.6 (2)
C18—C8—C9—C11	66.0 (2)	C12—C13—C17—C16	−149.2 (2)
C14—C8—C9—C11	−55.6 (2)	C14—C13—C17—C16	−24.7 (2)
C7—C8—C9—C10	50.8 (3)	C15—C16—C17—C20	122.2 (2)
C18—C8—C9—C10	−68.0 (2)	C15—C16—C17—C13	−3.4 (2)
C14—C8—C9—C10	170.35 (18)	C13—C17—C20—O2	62.8 (3)
C2—C1—C10—C19	−72.2 (2)	C16—C17—C20—O2	−56.7 (2)
C2—C1—C10—C5	52.7 (2)	C13—C17—C20—C21	−54.3 (3)
C2—C1—C10—C9	165.77 (19)	C16—C17—C20—C21	−173.8 (2)
C6—C5—C10—C19	−63.9 (3)	C13—C17—C20—C22	−179.2 (2)
C4—C5—C10—C19	69.0 (3)	C16—C17—C20—C22	61.4 (3)
C6—C5—C10—C1	175.91 (17)	O2—C20—C22—C23	177.3 (3)
C4—C5—C10—C1	−51.2 (2)	C21—C20—C22—C23	−67.0 (3)
C6—C5—C10—C9	61.0 (2)	C17—C20—C22—C23	58.8 (3)
C4—C5—C10—C9	−166.10 (18)	C20—C22—C23—C24A	−80.0 (6)
C11—C9—C10—C19	−62.2 (3)	C20—C22—C23—C24	−93.2 (4)
C8—C9—C10—C19	70.3 (3)	C24A—C23—C24—C25	−12.1 (11)
C11—C9—C10—C1	56.5 (2)	C22—C23—C24—C25	134.1 (8)
C8—C9—C10—C1	−170.92 (19)	C23—C24—C25—C27	−0.7 (15)
C11—C9—C10—C5	171.24 (18)	C23—C24—C25—C26	−179.4 (9)
C8—C9—C10—C5	−56.2 (2)	C22—C23—C24A—C25A	125.3 (8)
C8—C9—C11—C12	55.7 (3)	C24—C23—C24A—C25A	165 (2)
C10—C9—C11—C12	−169.78 (18)	C23—C24A—C25A—C26A	−177.2 (9)
C9—C11—C12—C13	−56.3 (3)	C23—C24A—C25A—C27A	−2.7 (14)
C11—C12—C13—C17	−178.5 (2)		

Symmetry code: (i) −*x*+2, −*y*, *z*.

### Hydrogen-bond geometry (Å, º)

**Table:**

*D*—H···*A*	*D*—H	H···*A*	*D*···*A*	*D*—H···*A*
O2—H1*O*2···O1*W*	0.84	2.02	2.816 (3)	157
O1*W*—H1*W*1···O1^ii^	0.84	1.94	2.783 (3)	175
O2*W*—H1*W*2···O2^iii^	0.83	1.89	2.718 (3)	177

Symmetry codes: (ii) *y*, −*x*+1, *z*+1/2; (iii) *x*, *y*, *z*+1.
